# PI3K and MAPK Pathways as Targets for Combination with the Pan-HER Irreversible Inhibitor Neratinib in HER2-Positive Breast Cancer and TNBC by Kinome RNAi Screening

**DOI:** 10.3390/biomedicines9070740

**Published:** 2021-06-28

**Authors:** Jangsoon Lee, Huey Liu, Troy Pearson, Toshiaki Iwase, Jon Fuson, Alshad S. Lalani, Lisa D. Eli, Irmina Diala, Debu Tripathy, Bora Lim, Naoto T. Ueno

**Affiliations:** 1 Section of Translational Breast Cancer Research, Morgan Welch Inflammatory Breast Cancer Research Program and Clinic, Department of Breast Medical Oncology, The University of Texas MD Anderson Cancer Center, Houston, TX 77030, USA; huey.liu@mdanderson.org (H.L.); TPearson@mdanderson.org (T.P.); tiwase@mdanderson.org (T.I.); JAFuson@mdanderson.org (J.F.); dtripathy@mdanderson.org (D.T.); 2 Puma Biotechnology, Inc., Los Angeles, CA 90024, USA; ALalani@pumabiotechnology.com (A.S.L.); leli@pumabiotechnology.com (L.D.E.); idiala@pumabiotechnology.com (I.D.)

**Keywords:** neratinib, HER2-positive breast cancer, triple-negative breast cancer, RNAi screening, synergistic effect

## Abstract

Human epidermal growth factor receptor (EGFR) 2 (HER2) is overexpressed/amplified in about 25% of all breast cancers, and EGFR is overexpressed in up to 76% and amplified in up to 24% of triple-negative breast cancers (TNBC). Here, we aimed to identify inhibitors that may enhance the anti-tumor activity of neratinib for HER2+ breast cancer and TNBC. By conducting a non-biased high-throughput RNA interference screening, we identified PI3K/AKT/mTOR and MAPK as two potential inhibitory synergistic canonical pathways. We confirmed that everolimus (mTOR inhibitor) and trametinib (MEK inhibitor) enhances combinatorial anti-proliferative effects with neratinib under anchorage-independent growth conditions (*p* < 0.05). Compared to single agent neratinib, the combination therapies significantly enhanced tumor growth inhibition in both SUM190 HER2+ breast cancer (neratinib plus everolimus, 77%; neratinib plus trametinib, 77%; *p* < 0.0001) and SUM149 TNBC (neratinib plus everolimus, 71%; neratinib plus trametinib, 81%; *p* < 0.0001) xenograft models. Compared to single-agent neratinib, everolimus, or trametinib, both everolimus plus neratinib and trametinib plus neratinib significantly suppressed proliferation marker Ki67 and enhanced antitumor efficacy by activating the apoptosis pathway shown by increased Bim and cleaved-PARP expression. Taken together, our data justify new neratinib-based combinations for both HER2+ breast cancer and TNBC.

## 1. Introduction

The ERBB receptor tyrosine kinase family consists of four receptors: epidermal growth factor receptor (EGFR), human EGFR 2 (HER2), HER3, and HER4, which activate oncogenic pathways by homo- or heterodimerization in many types of cancers. EGFR is overexpressed in up to 76% and amplified in up to 24% of triple-negative breast cancers (TNBC) cases [1,2] and is correlated with a higher rate of recurrence, metastasis, and lower survival [3,4,5]. Several EGFR-targeted antibodies (e.g., cetuximab and panitumumab) combined with chemotherapy have been tested in clinical trials, in which they have improved the survival of patients with TNBC [6,7].

HER2 is a well-defined breast cancer biomarker and is overexpressed/amplified in about 25% of all breast cancers [8]. HER2-targeted antibody-based drugs (e.g., trastuzumab, pertuzumab, ado-trastuzumab emtansine, and fam-trastuzumab-deruxtecan-nxki) and small molecule kinase inhibitors (e.g., lapatinib, neratinib, and tucatinib) have been approved for the treatment of HER2+ breast cancer. All HER family members contribute to the behavior of breast cancer in various ways. HER2 and HER3 co-expression is associated with poor survival outcomes in patients with HER2+ breast cancer [9,10]. The co-existence of high levels of HER3 and EGFR is correlated with worse breast cancer-specific and distant metastasis-free survival for patients with TNBC [11]. HER4 is known to contribute to drug resistance and anti-apoptosis by dimerization with other ErbB receptors. In estrogen receptor-positive breast cancer, HER4 impairs the efficacy of tamoxifen and is associated with poor survival outcomes [12,13].

Neratinib (HKI-272), an orally administered small molecule, is an irreversible inhibitor of pan-ERBB receptor tyrosine kinases that acts by binding to the cysteine residue in the ATP-binding pocket of the receptor. Neratinib inhibits tyrosine kinase activity via binding to the ATP sites of these enzymes and prevents phosphorylation and activation of downstream signaling pathways. Neratinib is highly active in vitro against cell lines overexpressing EGFR or HER2 [14]. Neratinib also effectively inhibits the proliferation of EGFR- and HER2-expressing cells resistant to treatment with first-generation ERBB receptor tyrosine kinase inhibitors, such as gefitinib [15]. Neratinib was first approved by the U.S. Food and Drug Administration (FDA) for patients with early-stage HER2+ (overexpressed or amplified) breast cancer as an extended treatment after trastuzumab adjuvant therapy [16]. Subsequently, neratinib (combined with capecitabine) also received FDA approval to treat advanced or metastatic HER2+ breast cancer [17].

Given the clinical significance of the ERBB family in breast cancer and the clinical activity of neratinib, we conducted a preclinical study to identify kinase and pathway inhibitors that may enhance the anti-tumor activity of neratinib not only in HER2+ breast cancer but also in TNBC. Using high-throughput RNA interference (RNAi) kinome library screening, reverse phase protein array (RPPA), and conventional in vitro and in vivo preclinical models, we tested our hypothesis that neratinib with rational combination therapy can induce effective anti-tumor activity in both HER2+ breast cancer and TNBC.

## 2. Materials and Methods

### 2.1. Cell Lines and Reagents

MDA-MB-157, MDA-MB-231, MDA-MB-436, MDA-MB-453, MDA-MB-468, HCC1187, and HCC1806 cells were purchased from American Type Culture Collection (Manassas, VA, USA). HCC2185 and HCC3153 cells were purchased from UT Southwestern (Dallas, TX, USA). CAL51 and CAL120 cells were purchased from DSMZ-German Collection of Microorganisms and Cell Cultures GmbH (Braunschweig, Germany). SUM149, SUM159, SUM185, and SUM190 cells were purchased from Asterand Bioscience (Detroit, MI, USA). BCX010 and IBC3 cells were established at The University of Texas MD Anderson Cancer Center (Houston, TX, USA). FC-IBC02 cells were provided by Fox Chase Cancer Center (Philadelphia, PA, USA). KPL4 cells were provided by Kawasaki Medical School (Okayama, Japan). HCC1187, HCC1806, HCC2185, HCC3153, CAL51, and CAL120 cells were maintained in RPMI-1640 medium (#R7509, Sigma-Aldrich, St. Louis, MO, USA). MDA-MB-157, MDA-MB-231, MDA-MB-436, MDA-MB-453, and MDA-MB-468 cells were maintained in DMEM/F-12 medium (#D8062, Sigma-Aldrich). RPMI-1640 and DMEM/F-12 medium were supplemented with 10% fetal bovine serum (#F0900-050, GenDEPOT, Houston, TX, USA) and 1% antibiotic/antimycotic (Sigma-Aldrich). SUM149, SUM159, SUM190, FC-IBC02, BCX010, and IBC3 cells were maintained in Ham′s F-12 medium (#N6658, Sigma-Aldrich) and supplemented with 10% fetal bovine serum (#F0900-050, GenDEPOT), 1% antibiotic/antimycotic (#A5955, Sigma-Aldrich), 5 µg/mL insulin (#12585-014, Thermo Fisher, Waltham, MA, USA), and 1 µg/mL hydrocortisone (#H0888, Sigma-Aldrich). Neratinib was provided by Puma Biotechnology (Los Angeles, CA, USA), and everolimus, trametinib and olaparib were purchased from Selleck Chemicals (Houston, TX, USA). All listed cell lines were validated by short tandem repeat analysis at the Cytogenetics and Cell Authentication Core at the MD Anderson Cancer Center and confirmed to be free of mycoplasma.

### 2.2. In vitro Cell Proliferation Assay

The anti-proliferation effect of neratinib on cells was assessed using the CellTiter-Blue (#G8081, Promega, Madison, WI, USA) cell viability assay or sulforhodamine B staining assay. In brief, 1 to 6 × 10^3^ cells/well were seeded in 96-well plates and treated the next day with neratinib with or without target kinase inhibitors for 5 days. The CellTiter-Blue reagent was added to the plates, and the optical density at 595 nm was determined using a VICTOR X3 plate reader (PerkinElmer, Waltham, MA, USA). After completing the CellTiter-Blue assay, the cells were fixed with 5% trichloroacetic acid at room temperature for 2 h and then stained with a 0.025% sulforhodamine B solution (#AAA1476914, Fisher Scientific, Waltham, MA, USA) for 30 min at room temperature. After washing with 1% acetic acid solution to remove the excessive sulforhodamine B solution, the stained cells were dissolved in 10 mM Tris buffer. Optical density was determined fluorometrically at excitation and emission wavelengths of 488 and 585 nm, respectively, using the VICTOR X3 plate reader. Growth inhibition graphs were generated, and IG_50_s were calculated using nonlinear regression to fit the data to the log (inhibitor) vs. response (variable slope) curve using Prism software (V9.0, San Diego, CA, GraphPad, USA). The CalcuSyn program (V2, Biosoft, Cambridge, United Kingdom) was used to evaluate the synergistic anti-proliferation effect of neratinib in combination with kinase inhibitors.

### 2.3. High-Throughput RNAi Screening:

On day 1, we performed the reverse transfection of pooled siRNA. The kinome-pooled siRNAs, consisting of 3 unique siRNAs targeting 1 gene and a total of 2127 siRNAs targeting 709 kinase genes, were from Ambion Silencer Select Human Genome siRNA Library V4 (Life Technologies Inc., Carlsbad, CA, USA) and was loaded into a 384-well destination plate by an Echo 550 acoustic dispenser. For internal control, SilencerSelect Negative Control No. 1 siRNA (Thermo Fisher) and positive Silencer Select PLK1 siRNA (Thermo Fisher), as well as no-siRNA control, were included in each plate. Lipofectamine RNAiMAX (0.05 μL per well; Thermo Fisher) in 10 μL of serum-free Opti-MEM was added to each well of the destination plate using an EL-406 washer dispenser (Bio-Tek, Winooski, VT, USA). Then, the plate was centrifuged at 3000 rpm for 1 min, and the siRNA-RNAiMAX complex was incubated at room temperature for 45 min before 300 cells in 20 μL of complete medium without antibiotics was added by the EL-406 washer dispenser, yielding a total well volume of 30 μL and final siRNA concentration of 5 nM. The transfected destination plates were sealed with a breathable sealing membrane (#3345, Corning, Glendale, AZ, USA), and the cells were cultured for 48 h at 37 °C in 5% CO_2_ before the addition of neratinib. A total of 28 plates were reverse transfected for kinome screening. The destination plate map was as follows: no siRNA control, negative control siRNA, positive control siRNA, and 26 genes targeted by specific pooled siRNAs.

On day 3, we treated the samples with vehicle control (DMSO) and neratinib. Thirty microliters of DMSO or neratinib were individually added to each well by an EL-406 dispenser. The plates were sealed with a breathable sealing membrane and incubated for another 72 h for drug treatment.

On day 6, we conducted a viability assay. Thirty-five microliters of the medium in the screening plates was aspirated, and 25 μL of ATPlite 1step (PerkinElmer) was added in each well of the destination plate using an EL-406 dispenser. The plates were sealed with aluminum films (Sigma-Aldrich) and incubated at room temperature for 10 min while being shaken at 1000 rpm on an orbital shaker. After the plates were centrifuged at 1000 rpm for 5 min, luminescence was measured using a PheraStar microplate reader (BMG Labtech, Cary, NC, USA). ATP readings were used as an indicator of overall cell viability. For data analysis, the ATP readings for each condition were averaged and normalized to the mean of DMSO-treated no-siRNA negative control in the same plate as relative viability. The effect of pooled siRNA itself on cell viability was ranked by relative viability. The positive control PLK1 siRNA was used to monitor the transfection efficiency. PLK1-positive control and no-siRNA control were used to calculate each plate’s z′-factor ($Z^{\prime }\mathrm{factor}=\frac{3\times \left(\sigma p+\sigma n\right)\;}{\left|\mu p\;-\;\mu n\right|}$, where σp is the standard deviation of the positive control, σn is the standard deviation of the negative control, μp is the mean of the positive control, and μn is the mean of the negative control). The median z′-factor was 0.58 for kinome screening. The sensitivity index was calculated for each gene after treatment with neratinib to identify synergistic drug and gene combinations [18].

### 2.4. Anchorage-Independent Colony Formation (Soft Agar Assay)

The soft agar assay is a good predictor of in vivo activity in that a positive result is a potential indicator of in vivo carcinogenesis. It is also the most stringent assay for detecting malignant transformation of cells. For each of the listed TNBC and HER2+ breast cancer cell lines, cells (2 to 5 × 10^3^ cells/well) in 2 mL of 0.4% agarose solution were overlaid onto the bottom agar layer (0.7%) in 12-well plates. The plates were incubated for 3 to 6 weeks with or without the drug, and colonies were stained with 200 µL of 3-(4,5-dimethylthiazol-2-yl)-2,5-diphenyltetrazolium bromide solution (2 mg/mL; #AAL1193906, Fisher Scientific) for 3 h at 37 °C in 5% CO_2_. According to the manufacturer’s instructions, stable colonies greater than 80 μm in diameter were counted using a GelCount system (Oxford Optronix, Milton, UK).

### 2.5. RPPA

A total of 1 to 2.5 × 10^5^ cells/well were seeded in 6-well plates and treated the next day with neratinib with or without target kinase inhibitors for 3 days. Cells were washed with cold phosphate-buffered saline, and cell lysate was collected using *M*-*PER* solution (#78501, Thermo Fisher, USA) containing freshly added protease and phosphatase inhibitors (#B14002, #B15002, Bimake, Houston, TX, USA). The cell lysate was centrifuged in a microcentrifuge at 13,000 rpm for 10 min at 4 °C, and then the supernatant was collected. A total of 30 μL of cell lysates (1 μg/uL) was denatured in 10 μL of 4X sample buffer (40% glycerol, 8% sodium dodecyl sulfate, 0.25 M Tris-HCL, pH 6.8, and 10% 2-mercaptoethanol) at 95 °C for 10 min. RPPA samples were stored at 80 °C. The RPPA assay was performed at the Functional Proteomics Reverse Phase Protein Array (RPPA) Core facility at MD Anderson Cancer Center. In brief, cell lysate samples were serially diluted two-fold for five dilutions (i.e., undiluted, 1:2, 1:4, 1:8, and 1:16) and arrayed on nitrocellulose-coated slides. Sample spots were then probed with 304 antibodies by a tyramide-based signal amplification approach and visualized by diaminobenzidine colorimetric reaction to produce stained slides. Stained slides were scanned on a Huron TissueScope scanner to produce 16-bit tiff images. Sample spots in tiff images were identified and their densities quantified by Array-Pro Analyzer. Relative protein levels for each sample were determined by interpolating each dilution curve produced from the densities of the five-dilution sample spots using a “standard curve” (SuperCurve) for each slide (i.e., for each antibody). Relative protein levels were designated as log2 values and normalized for protein loading, and transformed to linear values.

### 2.6. Western Blotting

TNBC cells were treated with DMSO or neratinib, everolimus, trametinib, or a combination of these drugs for different times. Total protein extracts were prepared using a cold M-PER reagent, including phosphatase and protease inhibitors. A total of 10 μg of each sample was resolved by NuPAGE 4–12% Bis-Tris Plus gel (#WG1403BOX, Thermo Fisher) and then transferred onto a polyvinylidene difluoride membrane (#1620177, Bio-Rad, Hercules, CA, USA). Overnight, membranes were incubated at a 1:1000 dilution with anti-pHER2 (#2243, 1:320, Cell Signaling), anti-HER2 (#2243, Cell Signaling), anti-EGFR (#sc-373746, Santa Cruz Biotechnology, Inc., Dallas, TX, USA), anti-pEGFR (#3777, Cell Signaling), anti-pAKT (#9271, Cell Signaling), anti-pERK (#4370, Cell Signaling), anti-pmTOR (#2974, Cell Signaling), anti-Bim (#2933, Cell Signaling), or beta-actin (#A5441, Sigma-Aldrich). The secondary antibodies used were horseradish peroxidase-conjugated IgG (1:10,000, Life Technologies Inc.) for chemiluminescence detection with ImageQuant LAS4000 (GE Healthcare, Chicago, IL, USA) and Alexa Fluor-conjugated IgG (Life Technologies Inc.) for fluorescence detection with Odyssey IR imaging system (LI-COR Biosciences, Lincoln, NE, USA).

### 2.7. Caspase 3/7 Activity Assay

Caspase-Glo 3/7 assay reagent (#G8090; Promega) was used to measure apoptosis in the presence of neratinib with and without combination drug candidates according to the manufacturer’s instructions. In brief, HER2+ breast cancer (1 × 10^4^) or TNBC (5 × 10^3^) cells were seeded into a 96-well plate and incubated overnight. Cells were then treated with neratinib, everolimus, trametinib, or a combination of the two for 16 h. The culture medium was removed, and then equal volumes of Caspase-Glo 3/7 assay buffer were added to the cells, and cells were incubated for 1 h at 37 °C. The luminescent intensity of each well was measured using a Victor X3 microplate reader (PerkinElmer).

### 2.8. Animal Studies

For the in vivo studies, 4- to 6-week-old female athymic nude mice (Envigo, Indianapolis, IN, USA) were housed under pathogen-free conditions and treated in accordance with the National Institute of Health guidelines. To establish breast cancer cell xenografts, cell suspensions (4 × 10^6^ cells/100 µL) in 50% Matrigel were injected into one site in the abdominal mammary fat pad of each mouse. When tumors were approximately 70 to 150 mm^3^, mice were randomly distributed into 6 groups (*n* = 10 mice) to achieve similar average tumor volume (100 mm^3^) and standard deviation across the groups. The treatment started on the same day and continued until the mice in the control group become moribund. For the neratinib and trametinib groups, 0.5% hydroxypropyl methylcellulose plus 0.1% Tween 80 was used, and for the everolimus groups, 30% propylene glycol plus 5% Tween 80 was used. Tumor volume [*V* = 0.5 × (*L* × *W*^2^)] was measured by caliper, and body weight was measured twice weekly. According to the following equation, the antitumor effect of treatments was evaluated by percent tumor growth inhibition (TGI): average tumor volume of the treatment group/average tumor volume of the control group ×100.

### 2.9. IHC Staining

TNBC cell xenograft tumor tissues were fixed in 10% neutral-buffered formalin and embedded in paraffin. The sections (5 μm thick) were deparaffinized in xylene for 2 min 3 times, rehydrated in graded alcohols for 1 min, and washed in distilled water. IHC staining was performed using the Lab Vision automated system (Thermo Fisher) through the MD Anderson Cancer Center Division of Surgery Histology Core. The slides were then incubated with anti-Ki-67 (#RM-9106, 1:100, Thermo Fisher), anti-pHER2 (#2243, 1:320, Cell Signaling), anti-HER2 (#APA342AA, Biocare Medical, Pacheco, CA, USA), anti-EGFR (#4267, 1:50, Cell Signaling), anti-pEGFR (#API300AA, Biocare Medical), anti-Bim (#2933, 1:200, Cell Signaling), and anti-cleaved poly(ADP-ribose) polymerase (PARP, #5625, 1:100, Cell Signaling). Immunostained slides were scanned using an Aperio AT2 slide scanner (Leica, Buffalo Grove, IL, USA) and captured at 20× magnification using ImageScope software (Leica Biosystems, Wetzlar, Germany). Then intensity was quantified by ImageJ software [19].

### 2.10. Statistical Analysis

Cell proliferation and colony formation rates were summarized with descriptive statistics (mean and standard deviation of the mean) for each treatment group. The two-tailed unpaired *t*-test was used for statistical analysis using GraphPad Prism software, with *p* < 0.05 considered significant. For each set of anti-tumorigenicity data, tumor volumes were summarized with descriptive statistics (mean and standard error of the mean). Comparisons between treatment groups at a time point were made using two-way ANOVA and multiple comparisons test.

## 3. Results

### 3.1. Kinome RNAi Screening Revealed That PI3K and MAPK Pathways Are Potential Targets for Combination with Neratinib

**Figure 1:**
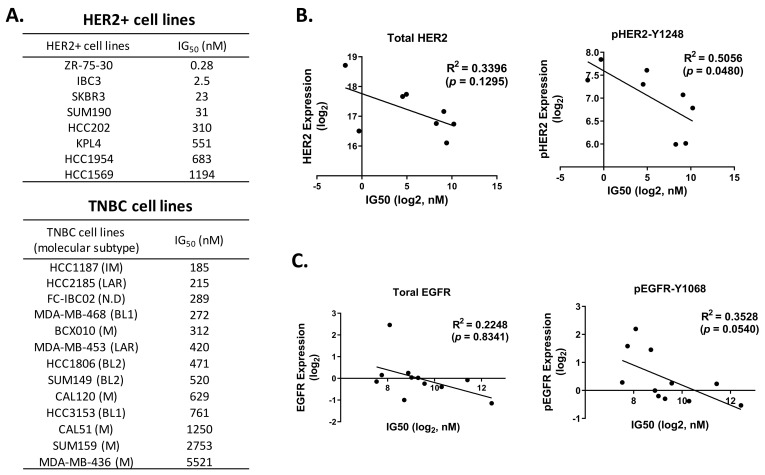
IG_50_ and phosphorylated ERBB expression levels in HER2+ breast cancer and TNBC cell lines were correlated. (**A**). Summary of IG_50_ of HER2+ breast cancer and TNBC cell lines. The anti-proliferation effect of neratinib was determined using CellTiter-Blue and sulforhodamine B staining at 5 days incubation with neratinib or DMSO control. Experiments were repeated in triplicate. BL1, basal-like 1; BL2, basal-like 2; HR; hormone receptor; IM, immunomodulatory; LAR, luminal androgen receptr; M, mesenchymal; N.D, not determined. (**B**,**C**). Inverse correlation between IG_50_ and pHER2 expression in HER2+ breast cancer cell lines (**B**) and inverse correlation between IG_50_ and pEGFR expression in TNBC cell lines (**C**). R values were calculated by Prism software.

To select the most appropriate HER2+ breast cancer and TNBC cell lines to conduct RNAi screening, we determined the anti-proliferation effect of neratinib in 8 HER2+ breast cancer and 13 TNBC cell lines. In vitro proliferation assays showed that neratinib had various ranges of IG_50_ in the tested HER2+ breast cancer (IG_50_ = 0.28–1194 nM, median IG_50_ = 171 nM) and TNBC (IG_50_ = 185–5521 nM, median IG_50_ = 471 nM) cell lines (Figure 1A and Supplementary Figure S1A,B). We did not observe any correlation between Vanderbilt TNBC molecular subtypes [20] and neratinib sensitivity. We next determined the correlation between ERBB protein activation/signaling and neratinib treatment sensitivity given neratinib’s pan-HER activity. Eight HER2+ breast cancer and 11 TNBC cell lines were analyzed using Western blotting and an RPPA database, respectively. The data revealed that the efficacy of neratinib was correlated with expression level of phosphorylated HER2 (pHER2; R^2^ = 0.5056, *p* = 0.0480) but not HER2 (R^2^ = 0.3396, *p* = 0.1295) across the HER2+ breast cancer cell lines tested (Figure 1B and Supplementary Figure S1C). In the tested TNBC cell lines, the efficacy of neratinib was correlated with expression level of phosphorylated EGFR (pEGFR, R^2^ = 0.3528, *p* = 0.0540) but not EGFR (R^2^ = 0.2248, *p* = 0.1406; Figure 1C). We found that pEGFR expression correlated with the expression level of pHER2 (R^2^ = 0.8341, *p* < 0.0001; Supplementary Figure S1D), and pHER2 expression, which also correlated with the IG_50_ of neratinib in TNBC cell lines (R^2^ = 0.3791, *p* = 0.0437; Supplementary Figure S1E). The IG_50_ of neratinib inversely correlated with pEGFR or pHER2 expression level, supporting neratinib’s on-target effect (Supplementary Figure S1F). These data indicated that phosphorylation levels of ERBB are highly predictive of the anti-proliferation effect of neratinib.

**Figure 2:**
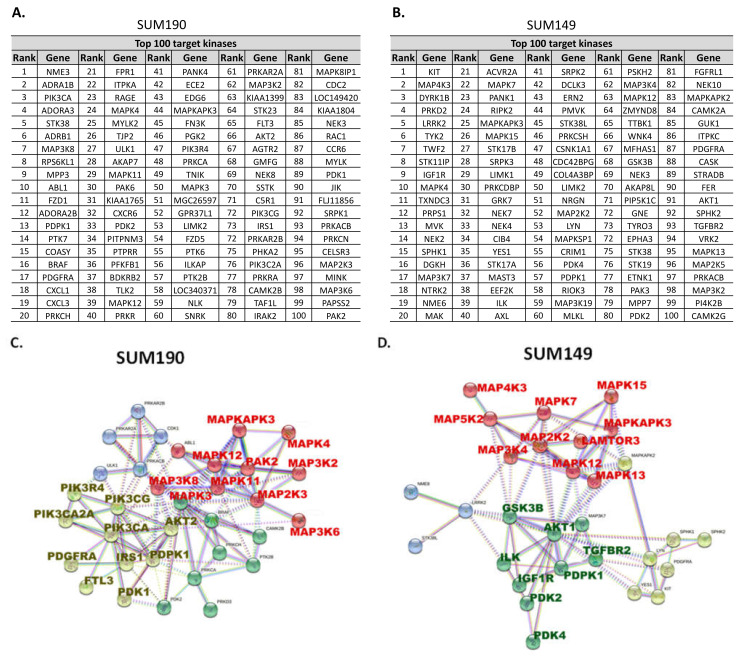
High-throughput siRNA library screening and protein-protein interaction analysis identified PI3K and MAPK pathways as potential candidates for combination with neratinib in TNBC and HER2+ breast cancer cell lines. (**A**,**B**). The top 100 target genes by sensitivity index analysis in SUM190 (**A**) and SUM149 (**B**) cells. (**C**,**D**). Canonical pathways identified by protein-protein interaction analysis and clustering (STRING V11) of SUM190 (**C**) and SUM149 (**D**) cells. Non-canonical target genes were excluded when exporting the image.

After defining the IG_50_ of neratinib, we selected HER2+ breast cancer cell line SUM190 and TNBC cell line SUM149 for RNAi screening because these cell lines showed near median IG_50_ concentration neratinib (Figure 1A) and demonstrated consistent tumorigenicity in nude mice. Using these cell lines, we performed a non-biased RNAi screening using a 709-kinase library to identify the target genes whose targeting synergistically inhibits cell viability in neratinib-treated cells. Following reverse RNAi transfection, cells were treated with DMSO control or an IG_30_ concentration of neratinib (10 nM for SUM190 and 100 nM for SUM149) for 3 days. We measured cell viability and then applied a sensitivity index to identify the top 100 potential targets that showed enhanced anti-proliferation activity of neratinib (Figure 2A,B).

Protein-protein interaction analysis (STRING, https://string-db.org/ accessed on 1 May 2021) [21] indicated that PI3KCA and MAPK3 (ERK1) are key target molecules for SUM190 TGI (Figure 2C) and that AKT1 and MAP2K2 (MEK2) are key target molecules for SUM149 TGI (Figure 2D). These molecules showed synergy with neratinib treatment among canonical pathway targets. These data indicated that PI3K/AKT and MAPK are potential target pathways that can synergistically inhibit HER2+ breast cancer and TNBC cell lines’ growth when combined with neratinib.

### 3.2. Everolimus (mTOR Inhibitor) and Trametinib (MEK Inhibitor) Enhance the Efficacy of Neratinib in Both TNBC and HER2+ Breast Cancer Cell Lines

**Figure 3:**
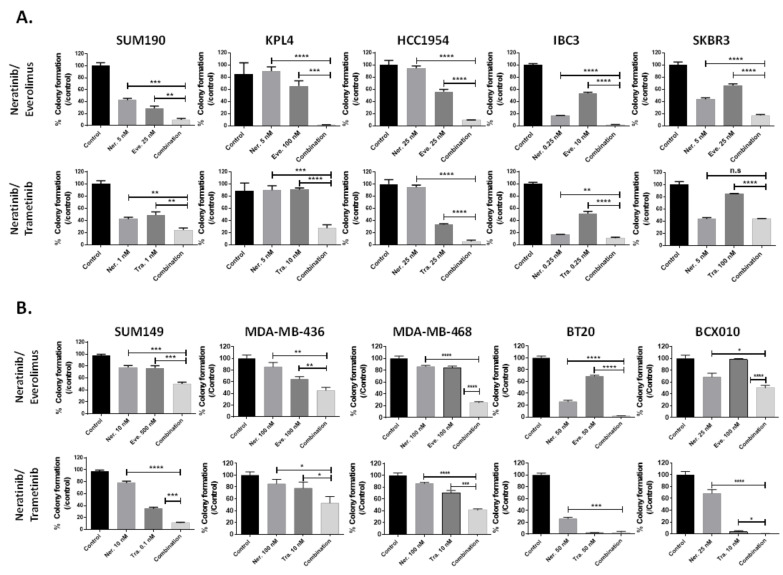
PI3K and MAPK pathway inhibitors enhanced the efficacy of neratinib in TNBC and HER2+ breast cancer cell lines. (**A**). Neratinib single-agent and combination therapy efficacy in HER2+ breast cancer cell lines. (**B**). Neratinib single-agent and combination therapy efficacy in TNBC cell lines. Each box shows mean ± s.d. n.s., not significant; *, *p* < 0.05; **, *p* < 0.01; ***, *p* < 0.001; ****, *p* < 0.0001. Experiments were repeated in triplicate.

To validate our RNAi screening findings, we selected the FDA-approved drugs, everolimus (to target the PI3K/AKT pathway) and trametinib (to target the MAPK pathway). We first determined the anti-proliferation effect of neratinib in combination with everolimus or trametinib in two-dimensional culture conditions. Neratinib plus everolimus showed the strongest anti-proliferation activity, with combination index values between 0.01 and 0.5 in 14 of the 16 cell lines (Supplementary Figure S2A). Trametinib combined with neratinib showed a moderate anti-proliferation effect, with combination index values between 0.2 and 0.9 for 9 of the 16 cell lines (Supplementary Figure S2B). Based on the proliferation assay results, we determined the in vitro anti-tumor effect of the neratinib combination in anchorage-independent growth conditions (i.e., a soft-agar assay). The number of colonies was significantly lower in cells treated with neratinib combined with either everolimus or trametinib than in cells treated with either everolimus or trametinib alone (*p* < 0.05; Figure 3A,B).

From non-canonical targets, we selected *BRCA* because about 20% of patients with TNBC have *BRCA1* or *BRCA2* mutations, and PARP-targeted therapy is approved for patients with *BRCA*-mutated TNBC [22]. RPPA analysis showed that *BRCA*-mutated cells (HCC1937, HCC3153, or MDA-MB-436) had ERBB and heregulin expression levels similar to or higher than those in the *BRCA*-mutated SUM149 cell line (Supplementary Figure S2C). We tested the combined effect of neratinib and the PARP inhibitor olaparib in two *BRCA*-mutated and four *BRCA*-wild types TNBC cell lines. We observed a significantly enhanced anti-proliferation effect in *BRCA*-mutated TNBC cell lines only (Supplementary Figure S2D).

**Figure 4:**
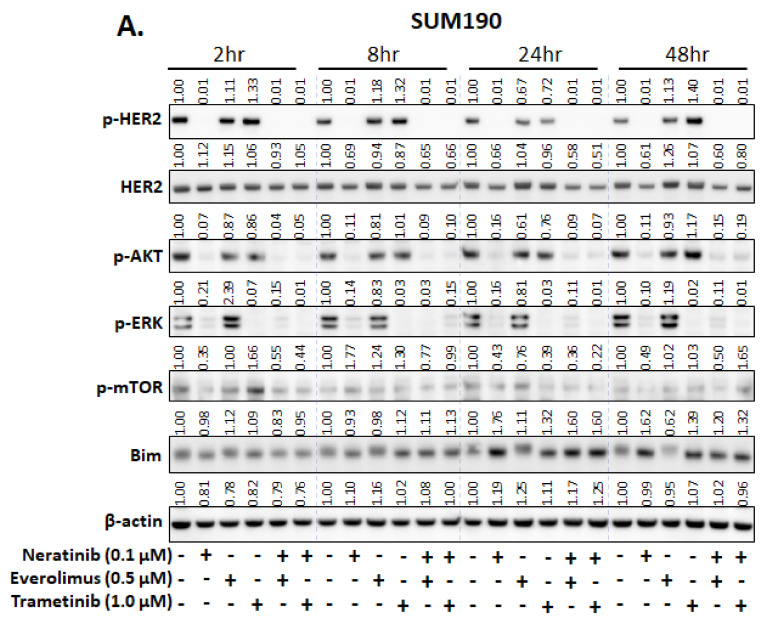

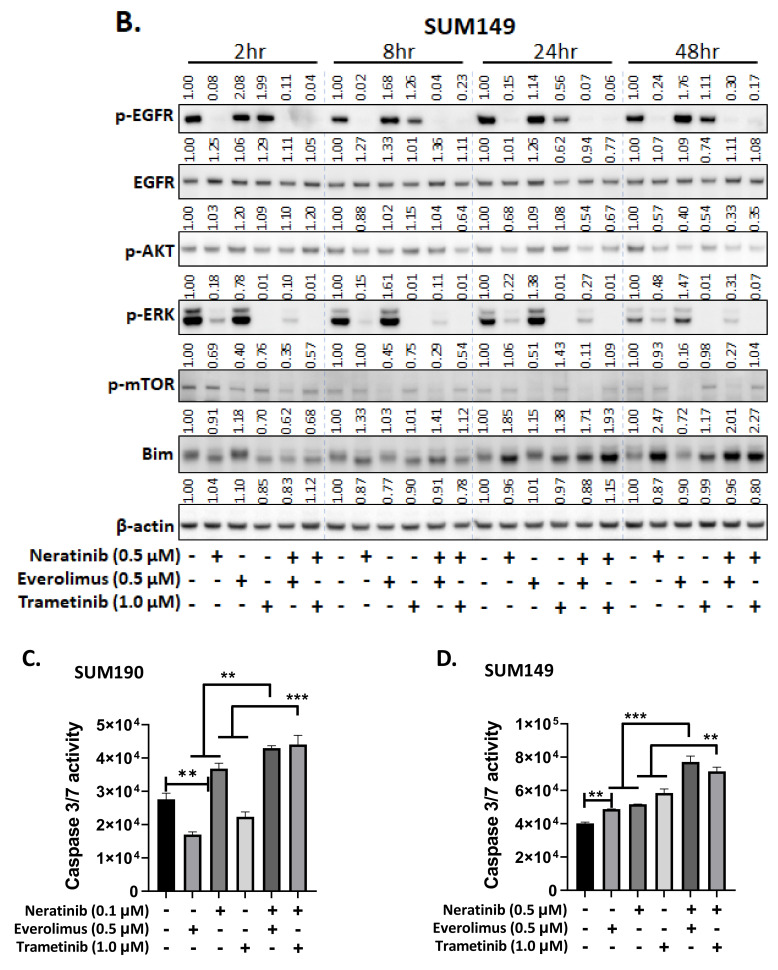
The combinatorial anti-proliferation effects of neratinib plus everolimus or trametinib were induced by apoptosis. (**A**,**B**). Western blotting results for SUM190 (**A**) and SUM149 (**B**) cells. (**C**,**D**). Caspase-Glo 3/7 assay of SUM190 (**C**) and SUM149 (**D**) cells. Cells were treated with the indicated drug for 16 h, and then caspase 3/7 activity was measured. Each box shows mean ± s.d; **, *p* < 0.01; ***, *p* < 0.001. Experiments were repeated in triplicate.

Next, to validate the specific target inhibition of neratinib, trametinib, and everolimus treatment, we analyzed the expression of proteins in the ERBB (EGFR, pEGFR, HER2, and pHER2), PI3K/AKT/mTOR (pAKT and pmTOR), and MAPK (pERK) pathways by Western blotting. In HER2+ breast cancer SUM190 cells, neratinib treatment significantly downregulated pHER2, pAKT, pERK, and pmTOR in a time-dependent manner (Figure 4A), whereas in TNBC SUM149 cells, neratinib treatment significantly inhibited pEGFR and pERK (Figure 4B). No additional inhibitory effect was observed when neratinib was combined with everolimus or trametinib.

RPPA data showed that neratinib treatment induced a significant increase in Bim expression in neratinib-sensitive HER2+ breast cancer and TNBC cell lines (Supplementary Figure S3A,B). Since Bim is an important apoptotic regulator and plays a key role in the effect of ERBB-targeting drugs in cancer cells [23,24], we evaluated the neratinib treatment-induced change of Bim expression in SUM190 and SUM149 cell lines. As a single agent, neratinib significantly increased Bim expression in both cell lines in a time-dependent manner (Figure 4A,B). No additional increase was observed when neratinib was combined with everolimus or trametinib. Still, BIM expression was sustained in single neratinib treatment (Figure 4A,B). Based on proliferation and Western blot data, we expected to see increased cell death in cells treated with neratinib alone or with neratinib plus everolimus or trametinib. We measured caspase 3/7 activity to assess apoptosis under drug treatment conditions to validate our observation. In the SUM190 HER2+ breast cancer cell line, only neratinib increased caspase 3/7 activity as a single agent compared to the control (*p* < 0.01) (Figure 4C). With everolimus and trametinib as single agents, caspase 3/7 activity was in fact lower than it was under the control condition. We speculate that this lower caspase 3/7 activity was due to the activation of compensatory signaling. Support for this interpretation comes from our observations that at early timepoints, SUM190 cells treated with everolimus exhibited elevated pERK expression compared to control and SUM190 cells treated with trametinib exhibited elevated p-mTOR expression compared to the control (Figure 4A). It is known that the MAPK pathway is one of the major resistant mechanisms of everolimus [25]. AKT/mTOR pathway can induce resistance to MEK inhibitor [26]. For that reason, we speculate that single treatment of everolimus or trametinib have lower caspase 3/7 activity than control. The combination of neratinib plus everolimus or neratinib resulted in significantly increased caspase 3/7 activity compared to single-agent neratinib (*p* < 0.01, neratinib and everolimus; or *p* < 0.001, neratinib and trametinib; Figure 4C). In the SUM149 TNBC cell line, single treatments slightly increased caspase 3/7 activity (neratinib, everolimus, or trametinib, *p* < 0.01), but more importantly, combination treatments significantly increased caspase 3/7 activity compared to single agent treatment (*p* < 0.001, neratinib and everolimus; *p* < 0.01, trametinib; Figure 4D). Similar results were observed using the HER2+ breast cancer HCC1954 and TNBC BCX010 cell lines (Supplementary Figure S4A,B). These results demonstrated that combination treatment with neratinib plus everolimus or trametinib significantly enhanced apoptosis in HER2+ breast cancer and TNBC cell lines compared to single-agent treatment with neratinib, everolimus, or trametinib.

### 3.3. Everolimus and Trametinib Enhance the Anti-Tumor Activity of Neratinib in Xenograft Models

**Figure 5:**
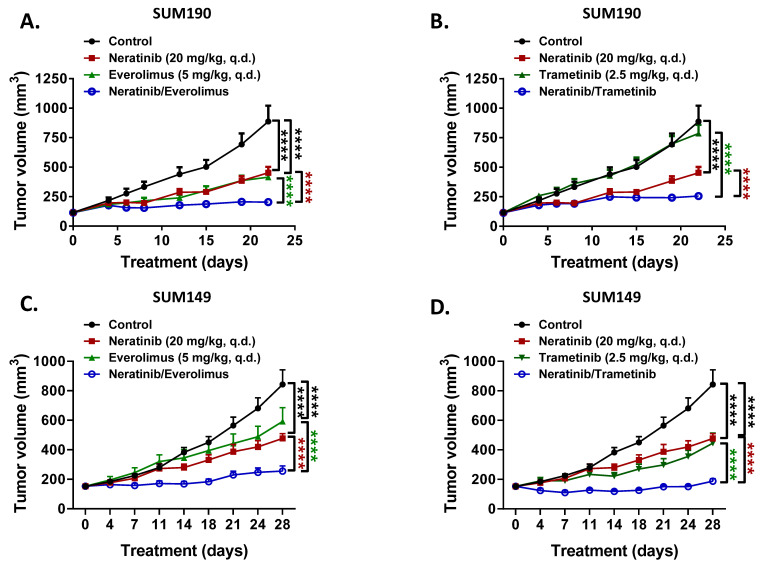

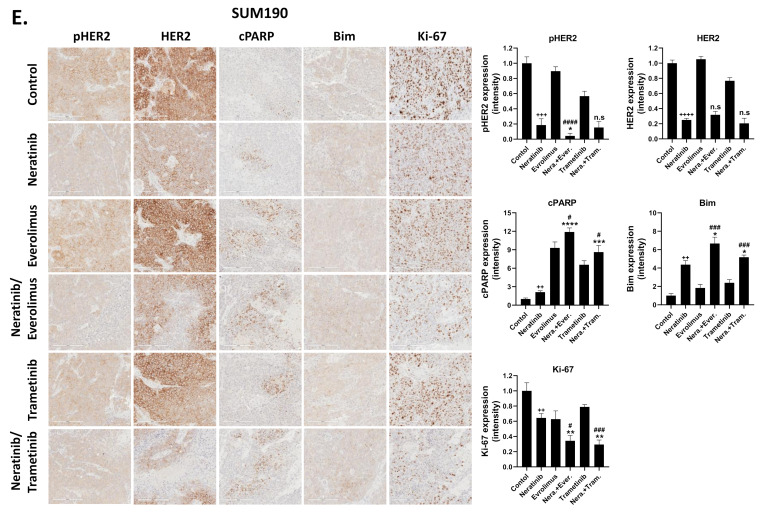

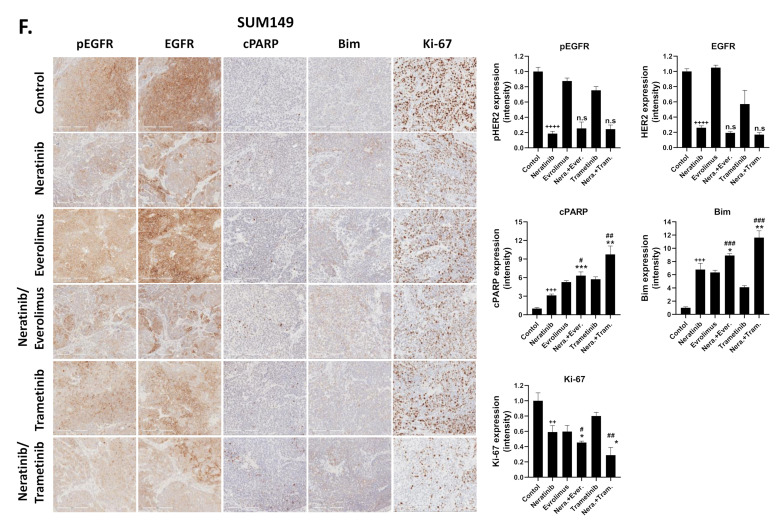
Everolimus or trametinib enhanced the anti-tumor effect of neratinib in both TNBC and HER2+ breast cancer cell line xenograft models. Mice were treated with neratinib (20 mg/kg), trametinib (2.5 mg/kg), everolimus (5 mg/kg), or a combination of two of the three daily by oral gavage (on a five day on and two day off schedule). (**A**,**B**). SUM190 (HER2+) xenograft model. (**C**,**D**). SUM149 (TNBC) xenograft model. The mice were treated with the indicated drugs for 22 days in the SUM190 xenograft model or 28 days in the SUM149 xenograft model. Each dot shows mean ± s.e.m. (10 mice/group). **** *p* < 0.0001. (**E**). IHC images of expression levels of pHER2, HER2, cPARP, Bim, and Ki-67 in SUM190 xenograft tumors tissues. (**F**). IHC images of expression levels of pEGFR, EGFR, cPARP, Bim, and Ki-67 in SUM149 xenograft tumors tissues. Data shown are representative of three IHC staining experiments from each treatment group with similar results. 20× magnification. Scale bars, 200 μm. Each box shows mean ± s.d. n.s., not significant; ++ *p* < 0.01, +++ *p* < 0.001, ++++ *p* < 0.0001 for control vs. neratinib; * *p* < 0.05, ** *p* < 0.01, *** *p* < 0.001, **** *p* < 0.0001 for neratinib alone vs. combination with everolimus or trametinib; ^#^ *p* < 0.05, ^##^ *p* < 0.01, ^###^ *p* < 0.001, ^####^ *p* < 0.0001 for everolimus or trametinib alone vs. combination with neratinib by unpaired *t* test.

Next, we evaluated the anti-tumor activity of neratinib as a single agent and in combination with everolimus or trametinib in SUM190 HER2+ breast cancer and SUM149 TNBC xenograft models. The doses were 20 mg/kg for neratinib, 5 mg/kg for everolimus, and 2.5 mg/kg for trametinib. Compared with the vehicle control, neratinib as a single agent showed a significant TGI effect in both SUM190 (Figure 5A, 49% TGI, *p* < 0.0001) and SUM149 (Figure 5C, 39% TGI, *p* < 0.0001) xenograft models. Everolimus as a single agent also showed a significant TGI effect compared with the vehicle control in both SUM190 (Figure 5A, 53% TGI, *p* < 0.0001) and SUM149 (Figure 5C, 35% TGI, *p* < 0.0001) xenograft models. Trametinib showed a significant TGI effect as a single agent in both SUM190 (Figure 5B, 11% TGI, *p* = 0.055) and SUM149 (Figure 5D, 47% TGI, *p* < 0.0001) xenograft models. For the combination of everolimus with neratinib, TGI was significantly increased in SUM190 (Figure 5A, 77% TGI, *p* < 0.0001) and SUM149 (Figure 5C, 71% TGI, *p* < 0.0001) compared to neratinib or single everolimus treatment. For trametinib plus neratinib, TGI was also significantly increased in SUM190 (Figure 5B, 77% TGI, *p* < 0.0001) and SUM149 (Figure 5D, 81% TGI, *p* < 0.0001) compared to single agent neratinib or trametinib treatments. Individual tumor-bearing mice showed consistent growth inhibition within a group divided by each treatment (Supplementary Figure S5A,B). There was no bodyweight loss in the mice treated with neratinib or in all the combinations during the treatment period (Supplementary Figure S5C), supporting the safety of neratinib treatment dose and schedule.

Next, we analyzed the expression level of pHER2, HER2, pEGFR, EGFR, Ki-67, cleaved PARP (cPARP), and Bim in tumor tissue samples by immunohistochemistry (IHC) staining (Figure 5E,F). We observed that the levels of HER2 and pHER2 in SUM190, and EGFR and pEGFR in SUM149 were significantly reduced in tumors from mice treated with neratinib alone (*p* < 0.001) but not in tumors from mice treated with everolimus or trametinib. We did not observe greater inhibition of HER2 and pHER2 in SUM190, and EGFR and pEGFR in SUM149 from neratinib plus everolimus or neratinib plus trametinib than from neratinib alone (Figure 5E,F), but we did observe sustained inhibition of pHER2 and pEGFR with combination treatment compared to single-agent neratinib treatment (Figure 5E,F). The expression levels of the Ki-67 proliferation marker were significantly reduced by neratinib single-agent treatment (*p* < 0.01) and were further reduced significantly when neratinib was combined with everolimus or trametinib (*p* < 0.05) (Figure 5E,F). To further validate that the anti-tumor effect of neratinib-based combination treatments resulted from apoptosis, we examined the expression levels of cPARP and Bim. As expected, the expression levels of cPARP and Bim in both xenograft models were significantly increased by both combination treatments (*p* < 0.05) compared to neratinib, everolimus, or trametinib single-agent treatments (Figure 5E,F). These results suggest that neratinib exerts a synergistic anti-tumor effect when combined with everolimus or trametinib by inducing apoptosis in both HER2+ breast cancer and TNBC xenograft tumors.

## 4. Discussion

To the best of our knowledge, this present preclinical study is the first to show that neratinib can potentially induce TGI in both HER2+ breast cancer and TNBC when co-treated with inhibitors of the PI3K/Akt-mTOR or MAPK pathway. Our findings in HER2+ breast cancer were supported by a recent study by Zhao et al. [27]. We also reported the novel finding of the potential anti-tumor effect of neratinib in both in vitro and in vivo xenograft models. Moreover, in the proteomics analysis of potential predictors of response to neratinib, we identified Bim as a potential predictive response biomarker for neratinib sensitivity in both HER2+ breast cancer and TNBC.

High-throughput kinome RNAi screening is widely utilized to discover cellular signaling pathways that underlie cancer biology and identify target protein kinases for new treatment development. Protein kinases regulate signaling pathways that drive the hallmark of cancer, such as proliferation, survival, metabolism, metastasis, angiogenesis, DNA damage response, and evading immunity; thus, this systemic functional genomics study is useful for new target discovery and new drug development for cancer. Recent improvements in systems biology research have revealed that relying solely on static point-in-time genetic analyses to diagnose cancer subtypes will miss patients whose cancer is driven by aberrant signaling that is not associated with an actionable mutation. This is because static measurements of proteins or genetic mutations in fixed cells cannot effectively characterize a dynamic biological system, such as signal transduction, which involves a cascade of an estimated 1020 biochemical states and complex feedback loops. Thus, for patients with breast cancer who lack actionable genomic or proteomic mutations or for whom targeted therapies associated with an actionable mutation no longer work, an alternative “omic” approach to diagnosing their breast cancer subtype is required.

Here, we confirmed new strategies in both HER2+ breast cancer and TNBC cell lines, not only by using the on-target effect of ERBB family protein inhibition but also by suppressing the compensatory PI3K-Akt-mTOR and MAPK pathways. A previous study demonstrated that the suppression of the PI3K-Akt-mTOR pathway by a PI3K inhibitor resulted in activation of HER family receptors via activation of the MAPK signaling pathway [28]. Intriguingly, the studies by others also demonstrated a significant anti-tumor effect when the PI3K inhibitor was combined with anti-HER2 therapy. Neratinib has a synergistic antitumor effect with everolimus in *PIK3CA*-mutated HER2+ breast cancer patient-derived xenograft models and with trametinib in HER2-mutated HER2+ breast cancer patient-derived xenograft models [27]. Combination treatment with lapatinib and ipatasertib overcomes resistance to anti-HER2 therapy in *PIK3CA*-mutant HER2+ breast cancer cells [29]. Targeting both the ERBB family protein and the compensatory pathway is relevant because the interaction between the receptor proteins and the activated signaling pathway is a mechanism of acquired resistance to anti-HER2 therapy [30].

Our strategy of targeting both HER2 and a compensatory pathway was supported by the positive results of a previously conducted phase 3 clinical trial (BOLERO-3, NCT01007942) that evaluated the efficacy of the combination of an mTOR inhibitor and trastuzumab in patients with trastuzumab-resistant advanced breast cancer [31]. The study confirmed that treatment with an mTOR inhibitor plus trastuzumab and vinorelbine restored the trastuzumab sensitivity of trastuzumab-resistant advanced breast cancer and significantly prolonged progression-free survival compared to the survival of patients treated with placebo plus trastuzumab and vinorelbine; however, the toxicity profile precluded the adoption of this combination [31]. A trial is currently being conducted to add PI3K inhibitor therapy to trastuzumab and pertuzumab in advanced HER2+ breast cancer and *PIK3CA*-mutant breast cancer (NCT04108858). These treatment strategies should also be clinically explored for patients with TNBC because a significant anti-tumor effect via the inhibition of ERBB family proteins and their compensatory pathways was observed in our preclinical TNBC model.

Of note, the randomized phase 3 ExteNET trial, which evaluated the efficacy of neratinib treatment after neoadjuvant/adjuvant trastuzumab-based therapy, showed efficacy only in patients with estrogen receptor positive/HER2+ early-stage breast cancer [32]. The result indicated the importance of suppressing both the compensatory pathway and the HER2 receptor signaling pathway to obtain efficacy in patients for whom HER2 targeted therapy produced an insufficient response [16]. Our preclinical model also showed an anti-tumor effect by suppressing both ERBB family proteins and compensatory pathway molecules. Co-inhibition of ERBB family and other pathways could be clinically explored for patients with TNBC based on our pre-clinical data.

In the I-SPY 2 neoadjuvant trial, phospho-EGFR (Y1068, Y1173, or Y992), HER2 total, phospho-HER2 (Y1248), and phospho-Shc (Y317) were associated with pCR and recognized as predictive biomarkers for neratinib treatment in patients with breast cancer. [33]. We also identified that pHER2 Y1248 expression is correlated with the efficacy of neratinib in both HER2+ breast cancer and TNBC cell lines. However, the efficacy of neratinib in TNBC cell lines was closely associated with pEGFR Y1068 expression but not with pEGFR Y1178 expression. We speculated that in vitro culture conditions might not represent the patient tumor microenvironment, such as ERBB ligands or cytokines.

PARP inhibitors (e.g., olaparib, rucaparib, niraparib, and talazoparib) are only approved in germline *BRCA*-mutated breast cancers. However, recent clinical trials have indicated that a larger number of patients with breast cancer may be sensitive to PARP inhibitors because of other DNA-repair gene mutations, DNA-repair deficiency, and somatic *BRCA1* and *BRCA2* mutations. In the RIO trial (EudraCT 2014-003319-12), 69% of patients with primary TNBC had a homologous recombination DNA repair deficiency signature [34]. In the TBCRC 048 trial (NCT03344965), PARP inhibition by olaparib was an effective treatment for patients with metastatic breast cancer who had germline *PALB2* or somatic *BRCA1* or *BRCA2* mutations, but not *ATM* or *CHEK2* mutations alone [35]. These data indicated that PARP inhibitor treatment might be effective in a subset of patients with TNBC with germline or somatic mutation in DNA-repair genes (*ATM*, *ATR*, *BAP1*, *BARD1*, *BLM*, *BRIP1*, *CHEK1*, *CHEK2*, *CDK12*, *FANCA*, *FANCC*, *FANCD2*, *FANCF*, *MRE11A*, *NBN*, *PALB2*, *RAD50*, *RAD51C*, *RAD51D*, and WRN) [36]. Further retrospective analyses of genomic profiles will be required to identify patients with breast cancer who will benefit from PARP-based treatment.

## 5. Conclusions

In this study, we utilized high-throughput kinome RNAi screening to identify kinase targets that increase HER2+ breast cancer and TNBC cells’ sensitivity to neratinib treatment. We identified the PI3K and MAPK pathways as potential targets for combination with neratinib to enhance its anti-tumor effect. Taken together, our data support the development of novel rational combinatorial treatment strategies for patients with HER2+ breast cancer and TNBC, such as the strategies that are currently being developed in clinical trials (NCT02400476, NCT03101748). In addition to pHER2 and pEGFR, which were predictors of response to neratinib in HER2+ breast cancer and TNBC, Bim was noted to contribute to neratinib sensitivity, suggesting the induction of apoptosis as a mechanism of action in neratinib combination therapies. Our study provides a rationale to conduct a clinical validation trial that determines the clinical efficacy of newly identified combination therapies for patients with HER2+ breast cancer or TNBC. Taken together, our comprehensive preclinical data provide justification to develop new treatment strategies with neratinib for HER2+ breast cancer and TNBC.

## Supplementary Materials

The following are available online at https://www.mdpi.com/article/10.3390/biomedicines9070740/s1, Figure S1: Anti-proliferation effect of Neratinib in HER2+ breast cancer and TNBC cell lines, Figure S2: Everolimus, trametinib, and olaparib enhanced neratinib efficacy in HER2+ breast cancer and TNBC cell lines, Figure S3: Neratinib treatment induced proapoptotic proteins and inhibition of cell cycle-related proteins in neratinib-sensitive TNBC and HER2+ breast cancer cell lines, Figure S4: Everolimus or trametinib enhanced apoptosis in combination with neratinib, Figure S5: Tumor growth and bodyweight change in xenograft models.
