# Supplementary material for: PI3K and MAPK Pathways as Targets for Combination with the Pan-HER Irreversible Inhibitor Neratinib in HER2-Positive Breast Cancer and TNBC by Kinome RNAi Screening
Source: Biomedicines. 2021 Jun 28;9(7):740. doi: 10.3390/biomedicines9070740 (PMC8301343; doi:10.3390/biomedicines9070740)
Supplement: Supplementary file 1 [file biomedicines-09-00740-s001.zip › biomedicines-1196364-supplementary.pdf]

biomedicines

MDPI

Supplementary information

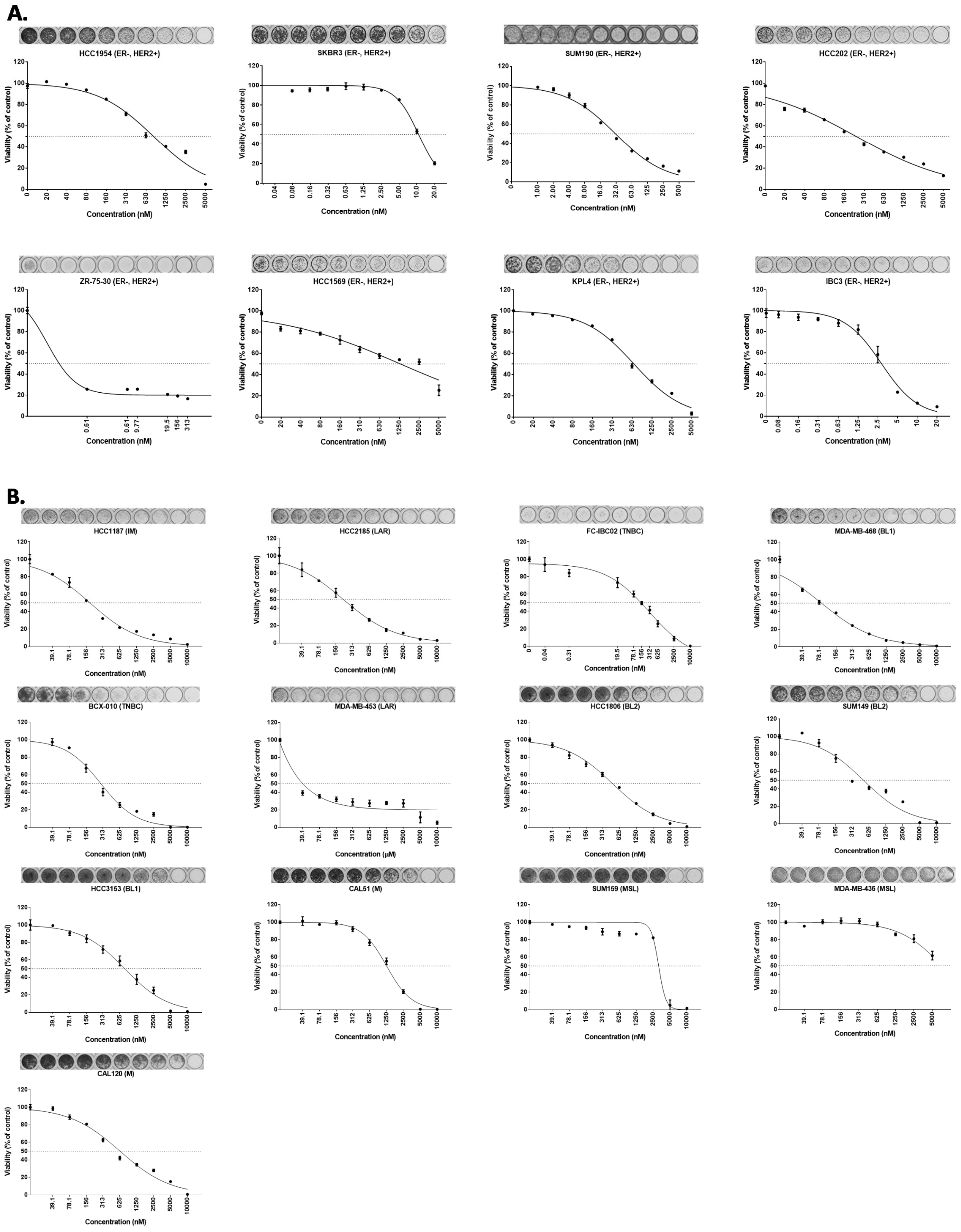

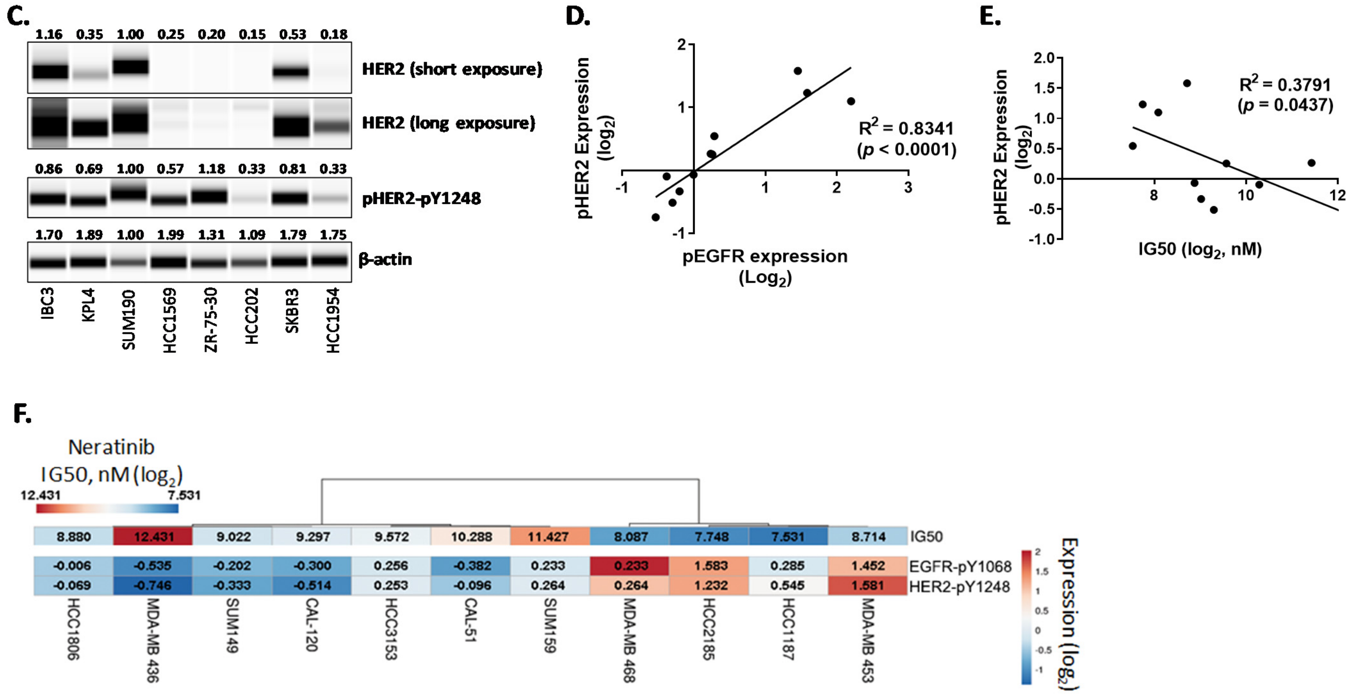

Figure S1. Anti-proliferation effect of Neratinib in HER2+ breast cancer and TNBC cell lines. Cells were treated with neratinib for 5 days. The growth curve was generated using GraphPad Prism software. Experiments were repeated in triplicate. A. HER2+ breast cancer cell lines. B. TNBC cell lines. C. Western blotting results for HER2+ breast cancer cell lines. D. TNBC cell lines showed a positive correlation between pEGFR and pHER2 expression. E. TNBC cell lines showed a negative correlation between IG_{50} and pHER2 expression. *R* values were calculated by Prism software. F. RPPA data of pEGFR and pHER2 in TNBC cell lines.

A.

Combinational index in neratinib/everolimus

HER2+

SKBR3			
Ner. (nM)	Eve. (µM)	Fa	CI
0.16	0.16	0.12	0.10
0.32	0.32	0.13	0.19
0.63	0.63	0.16	0.23
1.25	1.25	0.23	0.20
2.50	2.50	0.36	0.13
5.00	5.00	0.58	0.04

SUM190			
Ner. (µM)	Eve. (µM)	Fa	CI
0.04	0.16	0.82	0.11
0.08	0.32	0.84	0.14
0.16	0.63	0.88	0.10
0.32	1.25	0.92	0.06
0.63	2.50	0.94	0.06
1.25	5.00	0.96	0.03

HCC1954			
Ner. (µM)	Eve. (µM)	Fa	CI
0.04	0.16	0.63	0.03
0.08	0.32	0.68	0.05
0.16	0.63	0.73	0.08
0.32	1.25	0.79	0.11
0.63	2.50	0.85	0.16
1.25	5.00	0.90	0.20

HCC1569			
Ner. (µM)	Eve. (µM)	Fa	CI
0.04	0.16	0.45	0.10
0.08	0.32	0.48	0.11
0.16	0.63	0.53	0.10
0.32	1.25	0.59	0.10
0.63	2.50	0.64	0.13
1.25	5.00	0.64	0.25

HCC202			
Ner. (µM)	Eve. (µM)	Fa	CI
0.04	0.16	0.71	0.03
0.08	0.32	0.73	0.06
0.16	0.63	0.76	0.09
0.32	1.25	0.80	0.12
0.63	2.50	0.80	0.23
1.25	5.00	0.83	0.33

KPL4			
Ner. (µM)	Eve. (µM)	Fa	CI
0.04	0.16	0.31	0.03
0.08	0.32	0.47	0.03
0.16	0.63	0.66	0.04
0.32	1.25	0.85	0.03
0.63	2.50	0.92	0.04
1.25	5.00	0.96	0.04

IBC3			
Ner. (nM)	Eve. (µM)	Fa	CI
0.04	0.16	0.98	0.01
0.08	0.32	0.99	0.01
0.16	0.63	0.98	0.03
0.32	1.25	0.99	0.00
0.63	2.50	0.99	0.02
1.25	5.00	0.98	0.07

TNBC

MDA-MB-453			
Ner. (µM)	Eve. (µM)	Fa	CI
0.08	0.16	0.8	0.01
0.16	0.31	0.81	0.01
0.31	0.63	0.82	0.02
0.63	1.25	0.84	0.02
1.25	2.5	0.86	0.03
2.5	5	0.88	0.04

MDA-MB-468			
Ner. (µM)	Eve. (µM)	Fa	CI
0.08	0.16	0.14	> 1
0.16	0.31	0.27	> 1
0.31	0.63	0.41	0.09
0.63	1.25	0.53	0.11
1.25	2.5	0.75	0.14
2.5	5	0.84	0.22

SUM149			
Ner. (µM)	Eve. (µM)	Fa	CI
0.08	0.16	0.58	0.3
0.16	0.31	0.75	0.27
0.31	0.63	0.86	0.26
0.63	1.25	0.89	0.36
1.25	2.5	0.9	0.69
2.5	5	0.92	1.04

MDA-MB-436			
Ner. (µM)	Eve. (µM)	Fa	CI
0.04	0.16	0.36	0.11
0.08	0.31	0.4	0.06
0.16	0.63	0.41	0.06
0.31	1.25	0.48	0.01
0.63	2.5	0.59	0.01
1.25	5	0.67	0.01

BT-20			
Ner. (µM)	Eve. (µM)	Fa	CI
0.04	0.16	0.5	0.01
0.08	0.31	0.53	0.02
0.16	0.63	0.58	0.02
0.31	1.25	0.65	0.04
0.63	2.5	0.74	0.05
1.25	5	0.79	0.08

SUM159			
Ner. (µM)	Eve. (µM)	Fa	CI
0.16	0.16	0.49	0.11
0.31	0.31	0.59	0.17
0.63	0.63	0.74	0.23
1.25	1.25	0.8	0.38
2.5	2.5	0.89	0.51
5	5	0.99	0.26

BCX010			
Ner. (µM)	Eve. (µM)	Fa	CI
0.02	0.16	0.43	0.51
0.03	0.31	0.51	0.32
0.06	0.63	0.6	0.25
0.13	1.25	0.73	0.19
0.25	2.5	0.85	0.18
0.5	5	0.91	0.2

B.

Combinational index in neratinib/trametinib

HER2+

SKBR3			
Ner. (nM)	Tra. (µM)	Fa	CI
0.32	0.32	0.02	> 1
0.63	0.63	0.02	> 1
1.25	1.25	0.02	> 1
2.50	2.50	0.02	> 1
5.00	5.00	0.65	0.01
10.00	10.00	0.91	0.01

SUM190			
Ner. (µM)	Tra. (µM)	Fa	CI
0.04	0.16	0.71	0.53
0.08	0.32	0.79	0.33
0.16	0.63	0.86	0.16
0.32	1.25	0.92	0.07
0.63	2.50	0.93	0.08
1.25	5.00	0.92	0.22

HCC1954			
Ner. (µM)	Tra. (µM)	Fa	CI
0.04	0.16	0.27	0.103
0.08	0.32	0.35	> 1
0.16	0.63	0.59	> 1
0.32	1.25	0.82	> 1
0.63	2.50	0.88	> 1
1.25	5.00	0.87	> 1

HCC1569			
Ner. (µM)	Tra. (µM)	Fa	CI
0.04	0.16	0.33	0.18
0.08	0.32	0.40	0.18
0.16	0.63	0.51	0.16
0.32	1.25	0.59	0.16
0.63	2.50	0.64	0.23
1.25	5.00	0.65	0.40

HCC202			
Ner. (µM)	Tra. (µM)	Fa	CI
0.04	0.16	0.55	0.12
0.08	0.32	0.59	0.18
0.16	0.63	0.65	0.22
0.32	1.25	0.68	0.34
0.63	2.50	0.71	0.55
1.25	5.00	0.77	0.63

KPL4			
Ner. (µM)	Tra. (µM)	Fa	CI
0.04	0.16	0.10	0.07
0.08	0.32	0.10	0.10
0.16	0.63	0.10	0.04
0.32	1.25	0.10	0.15
0.63	2.50	0.18	0.10
1.25	5.00	0.41	0.08

IBC3			
Ner. (nM)	Tra. (µM)	Fa	CI
0.04	0.16	0.97	0.02
0.08	0.32	0.99	0.01
0.16	0.63	0.99	0.01
0.32	1.25	1.00	0.01
0.63	2.50	0.99	0.01
1.25	5.00	0.99	0.01

TNBC

MDA-MB-453			
Ner. (µM)	Tra. (µM)	Fa	CI
0.04	0.16	0.3	0.5
0.08	0.31	0.47	0.18
0.16	0.63	0.58	0.13
0.31	1.25	0.61	0.19
0.63	2.5	0.59	0.48
1.25	5	0.62	0.73

MDA-MB-468			
Ner. (µM)	Tra. (µM)	Fa	CI
0.04	0.16	0.11	> 1
0.08	0.31	0.14	> 1
0.16	0.63	0.27	> 1
0.31	1.25	0.41	0.42
0.63	2.5	0.53	0.49
1.25	5	0.75	0.36

SUM149			
Ner. (µM)	Tra. (µM)	Fa	CI
0.04	0.005	0.76	0.28
0.08	0.01	0.83	0.39
0.16	0.02	0.91	0.41
0.31	0.03	0.96	0.37
0.63	0.06	0.98	0.47
1.25	0.13	0.98	0.18

MDA-MB-436			
Ner. (µM)	Tra. (µM)	Fa	CI
0.04	0.16	0.01	> 1
0.08	0.31	0.01	> 1
0.16	0.63	0.01	> 1
0.31	1.25	0.03	> 1
0.63	2.5	0.1	> 1
1.25	5	0.21	> 1

BT-20			
Ner. (µM)	Tra. (µM)	Fa	CI
0.04	0.16	0.19	> 1
0.08	0.31	0.23	> 1
0.16	0.63	0.25	> 1
0.31	1.25	0.32	0.2
0.63	2.5	0.43	0.17
1.25	5	0.52	0.24

SUM159			
Ner. (µM)	Tra. (µM)	Fa	CI
0.16	0.16	0.43	0.96
0.31	0.31	0.63	0.33
0.63	0.63	0.68	0.43
1.25	1.25	0.77	0.52
2.5	2.5	0.82	0.77
5	5	0.999	0.03

BCX010			
Ner. (µM)	Tra. (µM)	Fa	CI
0.01	0.16	0.3	0.25
0.02	0.31	0.3	0.53
0.03	0.63	0.33	0.87
0.06	1.25	0.44	0.84
0.13	2.5	0.7	0.38
0.25	5	0.89	0.17

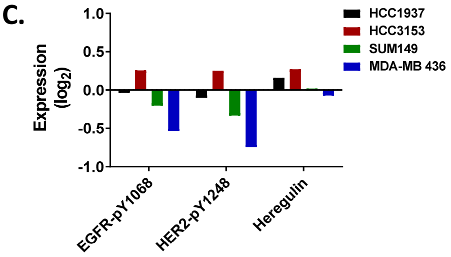

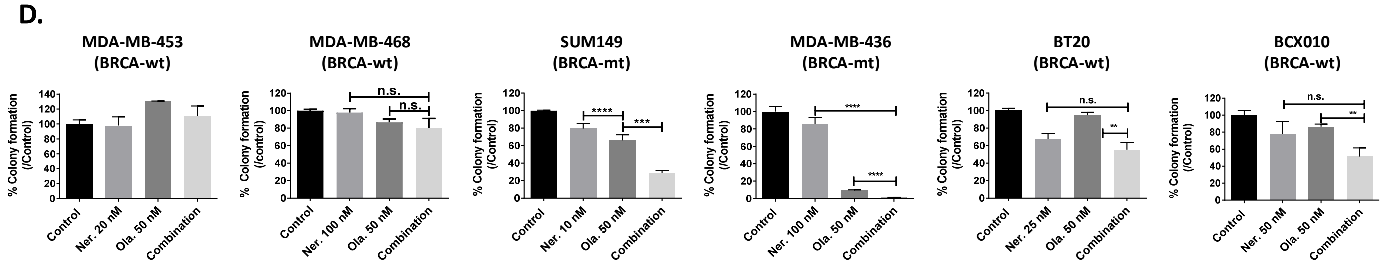

Figure S2. Everolimus, trametinib, and olaparib enhanced neratinib efficacy in HER2+ breast cancer and TNBC cell lines. A. Combination effect of everolimus with neratinib. B. Combination effect of trametinib with neratinib. Differential expression level of pEGFR, pHER2, and heregulin in *BRCA*-mutated TNBC cell lines. D. Combination effect of olaparib with neratinib in TNBC cell lines. Soft-agar assay. Each box shows mean ± s.d. n.s., not significant; **, $P < 0.01$; ***, $P < 0.001$; ****, $P < 0.0001$. Combinational index (CI) and fractional cell killing effect (Fa) were determined by using CalcuSyn software 2.1 (Biosoft). CI <0.1 indicates very strong synergism; 0.10-0.30, strong synergism; 0.31-0.70, synergism; 0.71-0.85, moderate synergism; 0.86-0.90, slight synergism; 0.91-1.10, nearly additive; 1.11-1.20, slight antagonism; 1.21-1.45, moderate antagonism; 1.46-3.30, antagonism; 3.31-10, strong antagonism; and >10, very strong antagonism. Experiments were repeated in triplicate.

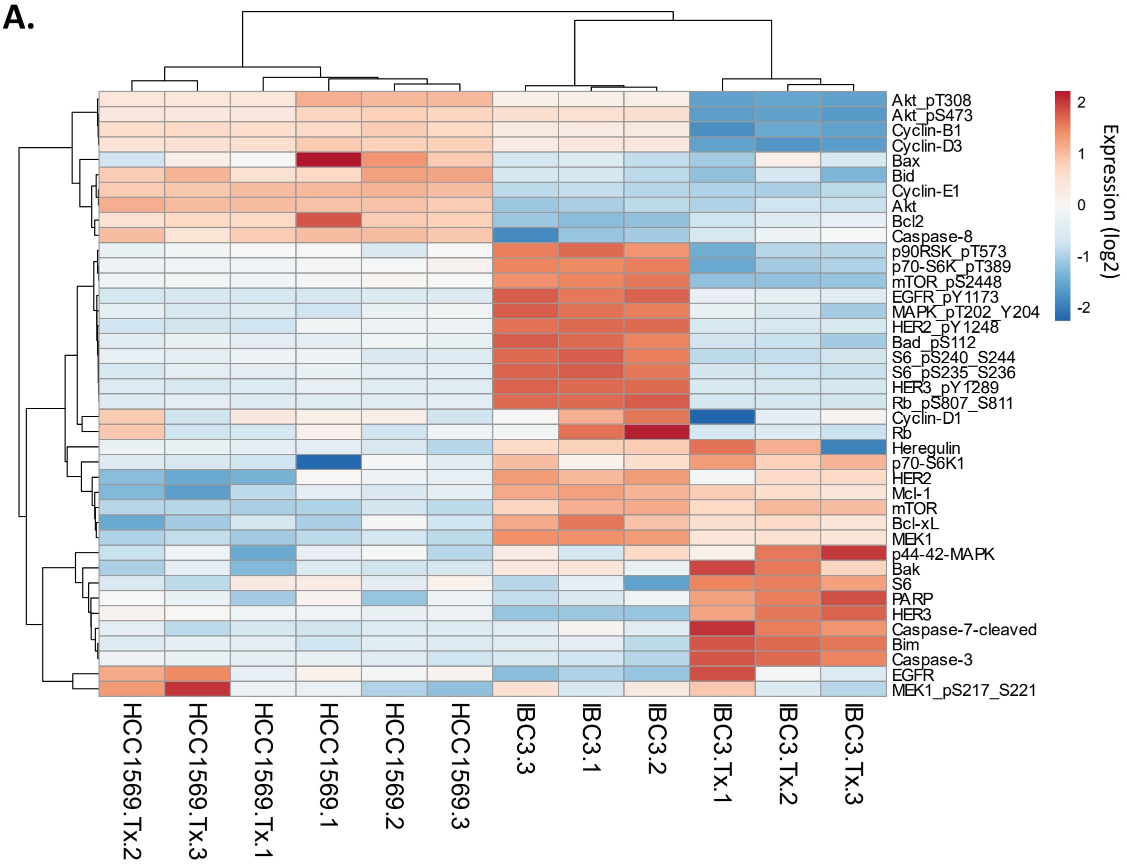

B.

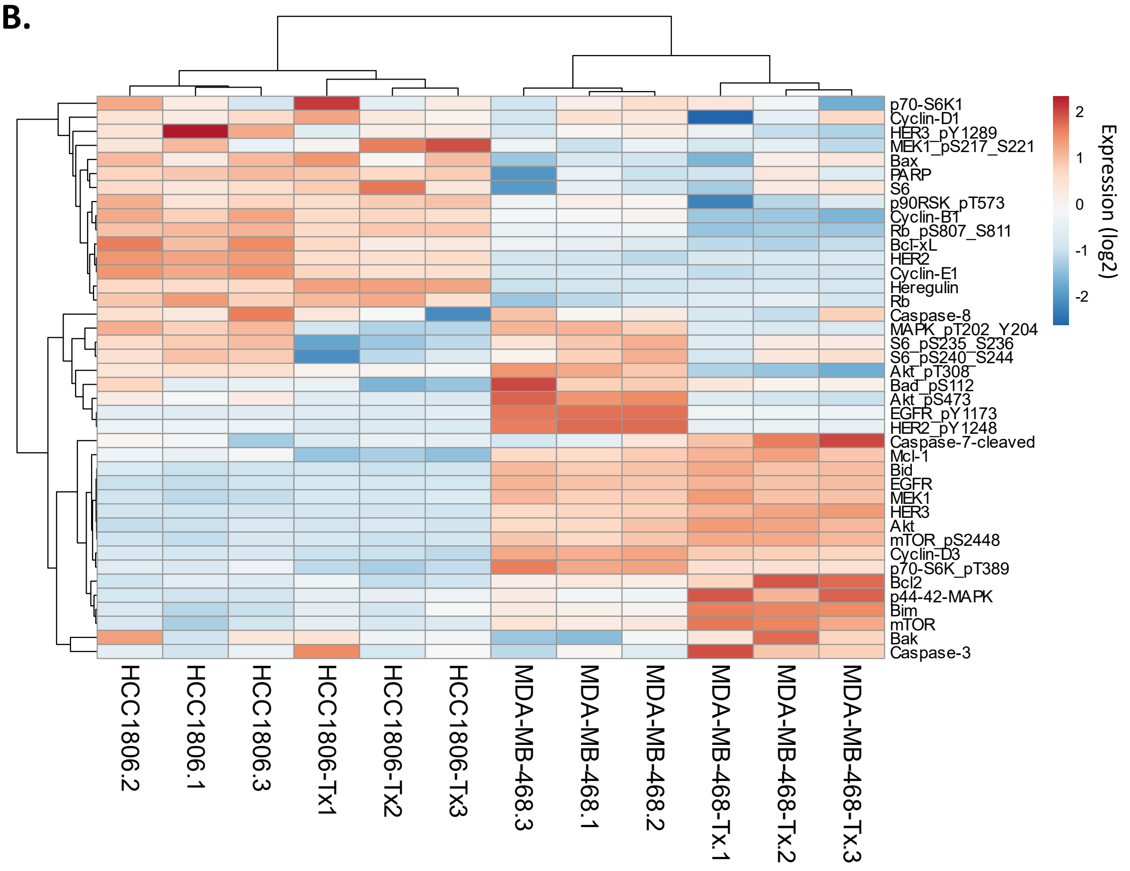

Figure S3. Neratinib treatment induced proapoptotic proteins and inhibition of cell cycle-related proteins in neratinib-sensitive TNBC and HER2+ breast cancer cell lines. 2×10^5 cells were plated into a 60-mm dish and incubated overnight. Cells were treated with or without neratinib (IBC3, 10 nM; HCC1569, 250 nM; MDA-MB-468, 250 nM; HCC1806, 250 nM) for 48 h, and cell lysates were collected for RPPA assay using M-PER mammalian protein extraction reagent. A and B. Heat maps of unsupervised hierarchical clustering and protein-protein interactome analysis of these identified proteins of RPPA. (A) IBC3 and HCC1569 HER2+ breast cancer cell lines. (B) MDA-MB-468 and HCC1806 TNBC cell lines.

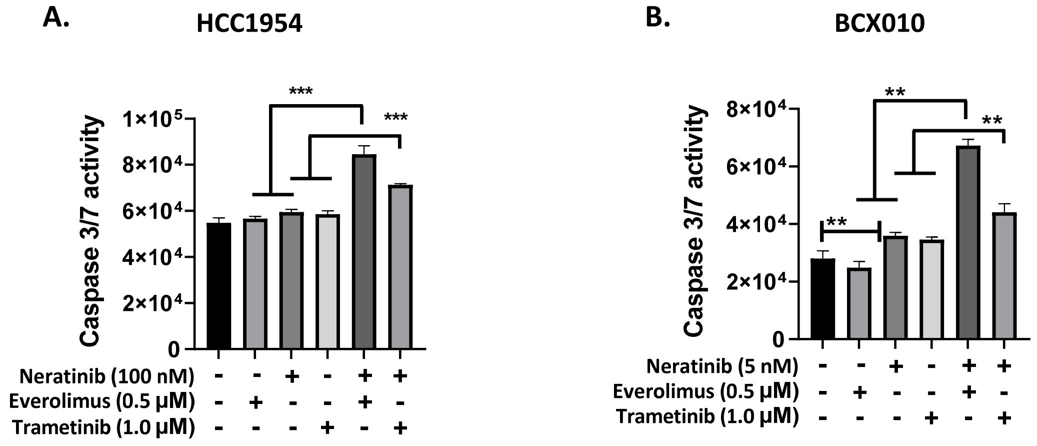

Figure S4. Everolimus or trametinib enhanced apoptosis in combination with neratinib. Caspase-Glo 3/7 assay was performed according to the manufacturer's instructions. A. HCC1954 HER2+ breast cancer cell line. B. BCX010 TNBC cell line. Each box shows mean ± s.d; **, $P < 0.01$; ***, $P < 0.001$. Experiments were repeated in triplicate.

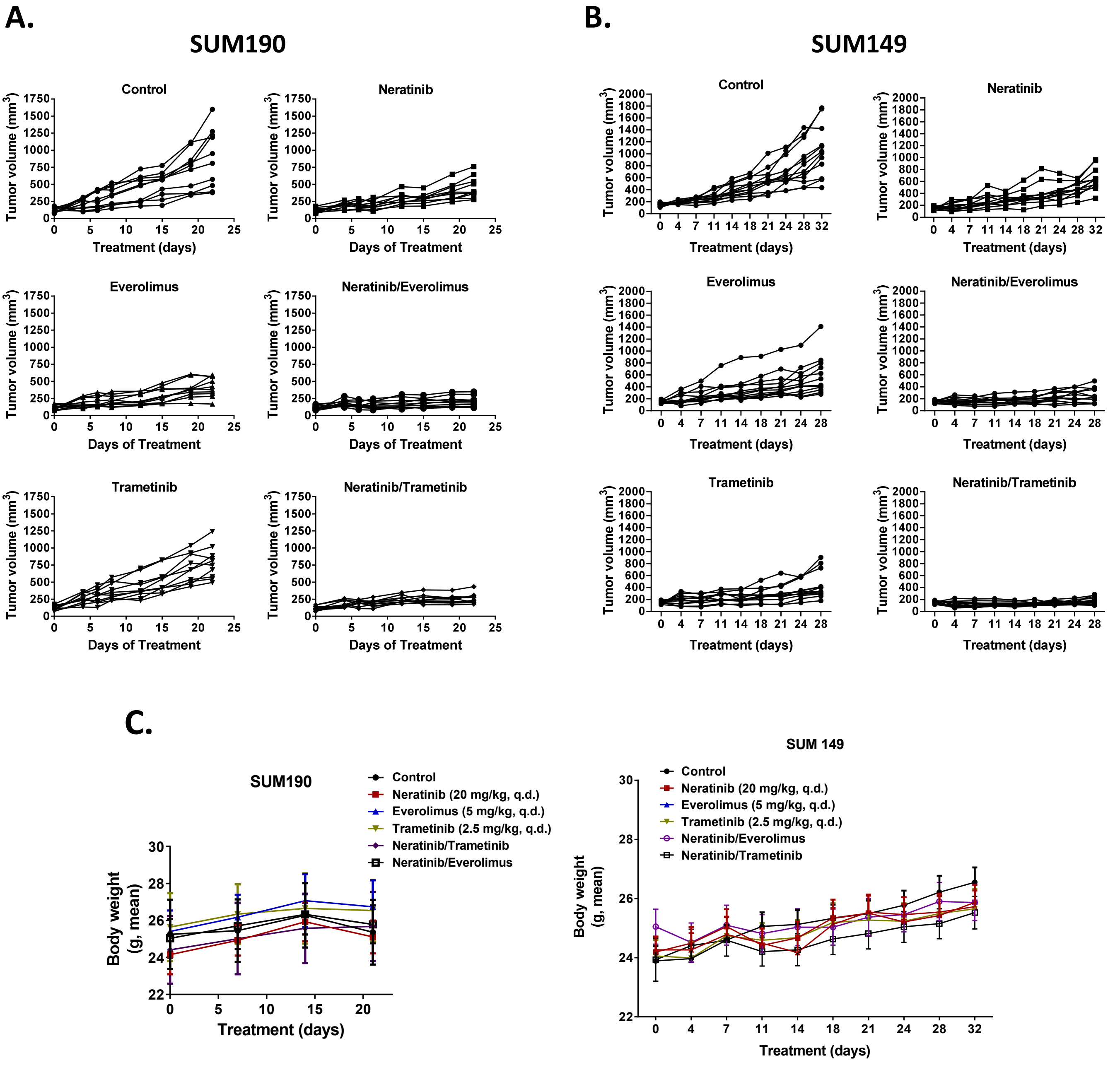

Figure S5. Tumor growth and bodyweight change in xenograft models. A. and B. Individual tumor growth in SUM190 (A) and SUM149 (B) models. C. Bodyweight change in the SUM190 and SUM149 xenograft models.
